# Identification of a self-paced hitting task in freely moving rats based on adaptive spike detection from multi-unit M1 cortical signals

**DOI:** 10.3389/fneng.2013.00011

**Published:** 2013-11-15

**Authors:** Sofyan H. H. Hammad, Dario Farina, Ernest N. Kamavuako, Winnie Jensen

**Affiliations:** ^1^Department of Health Science and Technology, Center for Sensory-Motor Interaction, Aalborg UniversityAalborg, Denmark; ^2^Department of Neurorehabilitation Engineering, Bernstein Center for Computational Neuroscience, University Medical Center Göttingen, Georg-August UniversityGöttingen, Germany

**Keywords:** motor cortex, micro-wire, cortical neuroprosthesis, wavelet, spike detection

## Abstract

Invasive brain–computer interfaces (BCIs) may prove to be a useful rehabilitation tool for severely disabled patients. Although some systems have shown to work well in restricted laboratory settings, their usefulness must be tested in less controlled environments. Our objective was to investigate if a specific motor task could reliably be detected from multi-unit intra-cortical signals from freely moving animals. Four rats were trained to hit a retractable paddle (defined as a “hit”). Intra-cortical signals were obtained from electrodes placed in the primary motor cortex. First, the signal-to-noise ratio was increased by wavelet denoising. Action potentials were then detected using an adaptive threshold, counted in three consecutive time intervals and were used as features to classify either a “hit” or a “no-hit” (defined as an interval between two “hits”). We found that a “hit” could be detected with an accuracy of 75 ± 6% when wavelet denoising was applied whereas the accuracy dropped to 62 ± 5% without prior denoising. We compared our approach with the common daily practice in BCI that consists of using a fixed, manually selected threshold for spike detection without denoising. The results showed the feasibility of detecting a motor task in a less restricted environment than commonly applied within *invasive BCI* research.

## INTRODUCTION

A brain–computer interface (BCI) aims to restore functional movements in subjects with neuromuscular disorders by interpreting neural signals recorded from the brain and translating the inferred information into control signals for external devices such as a prosthetic limb or to guide electrical stimulation of the patient’s own limbs (Taylor et al., 2002; Ethier et al., 2012; Vato et al., 2012). A BCI typically obtain neural information from electroencephalographic (EEG) signals recorded non-invasively with electrodes placed on the scalp. However, signals may also be recorded invasively from electrodes placed on the surface of the brain or microelectrodes implanted directly inside the brain tissue (Schwartz et al., 2006). These systems will be referred to as *invasive BCI* systems in the present work.

While an EEG signal represents the summation of the neural activity from thousands of neurons, intra-cortical microelectrodes detect the extracellular activity of a smaller neuronal population in the close vicinity of the recording site. Single-unit (SU) action potentials (APs), multi-unit (MU), or local field potential (LFP) recordings from the primary motor cortex (M1) have shown to encode specific information about limb kinematics, such as position (Amirikian and Georgopoulos, 2003), velocity (Reina et al., 2001), or muscle activity (Morrow and Miller, 2003), and also encode global information on the preparation of a movement (Donoghue et al., 1998).

In the past, the development of *invasive BCI* systems has mainly been driven by animal work and there has been a strong focus on predicting real-time, three-dimensional kinematic information for upper limb control using a center-out reaching task. For example, primates have learned to control reaching tasks of a robotic arm through information extracted from the motor cortex (Taylor et al., 2002; Carmena et al., 2003; Vargas-Irwin et al., 2010; Ifft et al., 2012). The neural encoding for this task is relatively well understood within these strict experimental paradigms. However, it is well known that the motor cortex encoding is dependent on visual, auditory, and somatosensory feedback (Salinas and Romo, 1998; Romo and Salinas, 2001). Therefore, an *invasive BCI* system must have the ability to decode the cortical signals reliably in a less controlled and ever changing environment (Mussa-Ivaldi and Miller, 2003; Musallam et al., 2004), and the availability of robust decoding algorithms is therefore essential. The decoding algorithms include methods for optimizing the signal-to-noise ratio (SNR) of the recordings, PA (or spike) detection, and the means to relate the cortical signals to motor tasks.

The majority of *invasive BCI*s today rely on detecting SU APs from individual neurons which requires sorting the spikes before decoding (see e.g., Laubach et al., 2000; Wessberg et al., 2000; Donoghue, 2002; Musallam et al., 2004; Hu et al., 2005; Olson et al., 2005; He, 2008; Truccolo et al., 2008). The spike sorting typically add processing time that is proportional to the number of detected neurons and number of recording channels (Ventura, 2008; Herzfeld and Beardsley, 2010). Furthermore, the number and characteristics of the detected spikes may be subjected to daily change due to small changes at the neural interface (e.g., micro-motions of the brain or fibrosis formation around the electrodes) or even cell death (Lewicki, 1998; Yu et al., 2012).

These drawbacks of using SU as input for decoding motor tasks recently led to the investigation of *invasive BCI* systems based on MU activity, which was found to provide either similar or greater decoding accuracy compared to the *invasive BCI* systems based on SU activities (Stark and Abeles, 2007; Fraser et al., 2009; Chestek et al., 2011). Multi-unit recordings are often associated with high levels of noise, originating from thermal, electrical, or biological sources, which necessitates improving the SNR prior to spike detection (Musial et al., 2002; Kim and McNames, 2007). Sources of non-stationary noise may also potentially hamper the performance of most filters. Linear, non-linear (Gozani and Miller, 1994; Franke et al., 2010), template matching (Paralikar et al., 2008), and Wiener filters have previously been used to improve the SNR of intra-cortical signals, however they are very sensitive to the noise properties (Oweiss and Anderson, 2001). Conversely, wavelets have been proven to perform well in the presence of non-stationary noise (Donoho, 1995; Oweiss and Anderson, 2001). The wavelet technique removes noise from the signal by thresholding the transformation coefficients in multiple frequency bandwidths. The use of a wavelet transformation requires the selection of a mother wavelet, which defines the decomposition filters and which is typically selected according to its similarity to the average shape of APs to be detected (Farina et al., 2007).

In multi-unit recordings, spikes may be detected by thresholding, template matching, or non-linear energy operators (Shalchyan et al., 2012). Thresholding is the most commonly used method due to the simplicity of its implementation (Rizk and Wolf, 2009), however it often requires human intervention to determine the threshold level (Nenadic and Burdick, 2005). Ideally, in complete autonomous systems such intervention must be minimized or completely eliminated. On the other hand, the use of unsupervised, adaptive methods for threshold determination does not require *a priori* knowledge of the noise levels and eliminates any threshold bias resulting from high firing rate or amplitudes in the data window compared to the threshold estimation using conventional methods (e.g., root-mean square or visual estimation; Chan et al., 2008). Adaptive thresholding has only been reported in few studies (see Quiroga et al., 2004; Hochberg et al., 2012), and has not been applied yet in multi-unit-based movement detection in freely moving animals.

Our main objective of this study was to investigate the possibility of detecting motor tasks from multi-units recordings in freely moving animals. The secondary objective was to analyze the influence of using adaptive thresholding with and without wavelet denoising on the accuracy of detecting the motor task. To evaluate the efficacy of our approach, we compared the results with the accuracy of detecting motor tasks based on manually selected thresholds for spike detection.

## MATERIALS AND METHODS

Four adult, male Sprague-Dawley rats were included in the study (age at inclusion time was 7 weeks, average weight of 200 g). All experimental procedures carried out was approved by the Animal Experiments Inspectorate under the Danish Ministry of Justice.

### ANIMAL BEHAVIORAL TASK

**FIGURE 1:**
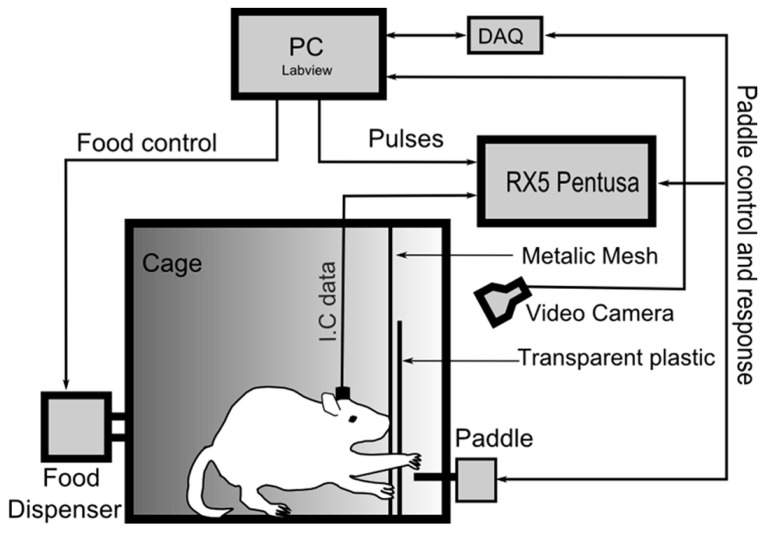
**Schematic overview of the experimental setup.** The animals were trained to hit a response paddle by reaching through a 1-cm-wide slot in a transparent wall placed in front of the paddle. During the workout, the intra-cortical signals were recorded using the multi-channel recording system (RX5 Pentusa), and the paddle response was streamed to LabView via a data acquisition card (DAQ).

Before the animals were implanted with cortical electrodes, we used standard psychophysical behavioral techniques to train the animals to reach their forelimb through a small opening to depress a response paddle in return for a food reward (see **Figure 1**). The restricted access to the paddle ensured that the rats rarely shifted between the preferred and non-preferred limb (visual observation).

The paddle lever (ENV-112CM, Med-Associates, Inc., USA) was placed at a height of approximately 6 cm from the bottom of the behavioral training cage. A transparent plastic wall was placed at a distance of approximately 2 cm in front of the paddle and had a 1-cm-wide by 7-cm-high opening. In addition, a platform was placed in the cage to allow the rats to support themselves while performing the hitting task. The food reward (Research Diets, Inc., New Brunswick, USA) was delivered with a pellet dispenser (ENV-203, Med-Associates, Inc., St. Albans, USA).

A paddle hit sequence was initiated by inserting the paddle into the cage. A successful sequence was defined as three consecutive hits (referred to as one trial) to exclude random events. After a successful trial, the paddle lever was automatically retracted for 9 s to allow time for the rats to eat the delivered reward. We thereby avoided possible interference of chewing artifacts and muscle activity with the recorded cortical signals. The animals were trained to hit the paddle as soon as it was protracted, but the hitting sequence was self-paced and the timing between each paddle hit was therefore not controlled. Each recording session continued until the rats had performed approximately 100 correct trials with their preferred limb.

In a number of cases, we defined the trials as failures, and they were discarded from further analysis. We excluded cases where the animals used their non-preferred forelimb paw and cases where they attempted to hit before the paddle lever was fully protracted. Finally, we excluded data where two consecutive paddle hits were less than 200 ms apart to avoid overlapping of the neural information encoding the individual hits. This resulted in a total of 1514 accepted trials across all rats, with a mean of 59 ± 21 trials per session. Only the first hit from every successful trial was selected for further data processing.

During the training and experiment sessions, the animals were kept food-restricted and were maintained on 90% of their normal body weight. Water was always provided *ad libitum*, and additional food was provided to maintain the targeted weight. The animals were housed in 12/12 h day/night cycle. The average training time was 3–4 weeks (30 min training sessions for 3–4 days per week).

### IMPLANT PROCEDURES

We used an anesthetic cocktail of ketamine (100 mg kg^-^^1^), xylazine (5 mg kg^-^^1^), and acepromazine (2.5 mg kg^-^^1^) in doses of 0.1 ml/100 g of body weight. A craniectomy was performed and the dura was removed over the primary motor cortex (M1) related to the forelimb movement contra-lateral to the preferred paw of each rat. An electrode array was implanted in the area related to forelimb movement (2 mm anterior and 2 mm lateral to Bregma at a depth of 1.6 mm; Deschênes et al., 1994; Jensen and Rousche, 2006). Two stainless steel bone screws were mounted on the skull 2–3 mm posterior and lateral to Bregma. The bone screw on the ipsilateral side of the electrode was used as a recording reference and the other was used for increasing the mechanical stability of the array by bridging the array and the screw with dental acrylic. After electrode implantation, the exposed brain was covered with collagen-based gel-foam (Johnson and Johnson, UK), and the electrode was fixed to the skull with dental acrylic (Heraeus, Germany).

The implanted electrodes were custom-made 4 × 4 tungsten micro-wire electrode arrays (2 × 2 mm, Teflon coated, diameter = 50 μm, length = 2–3 cm, spacing ~500 μm, A-M system, Inc., Sequim, USA). Each electrode shank was cut by laser to generate even surfaces to a total length of 0.5 cm. The wires were cut at 90° angle in relation to the longitudinal direction of the wires such that they had blunt tips. The laser used was a Ti:Sapphire amplified to 1 KHz beam at 800 nm, Tsunami, Spectra-Physics, Santa Clara, USA.

### DATA COLLECTION PROCEDURES

A total of 25 sessions were obtained from the four rats over an approximately 4-week period. The physiological signals and behavioral events were recorded using a multi-channel recording system (RX5 Pentusa, Tucker Davis Technology, Alachua, USA). The intra-cortical signals were band-pass filtered between 0.8 and 8 kHz and sampled at 24.414 kHz. The paddle events were simultaneously streamed to LabView through a data acquisition card (NI USB-6259 BNC, National Instruments, Austin, USA) to control the paddle lever (see **Figure 1**).

To assess the neural origin of the recorded intra-cortical activity, all rats were electrically stimulated after all recording were performed through the cortical electrodes (monophasic stimulus train = 100 Hz, pulse width = 200 μs, pulse amplitude ranged between 100 and 1500 μA). The stimulus was delivered at each channel, and the bone screw was used as the return reference. During this procedure, the animals were awake and were held by the experimenter in a fixed, prone position. The stimulus was delivered when the animal relaxed, did not move and the limbs were not supported. We mainly observed four different types of movements, which were categorized as “paw movements” (34%), “mixture of paw and neck movements” (19%), “neck movements” (11%), or “no response” (36%). Only the channels that showed paw movement response were selected for further data processing.

### DATA ANALYSIS

To analyze the influence of using adaptive thresholding for spike detection and wavelet denoising on the accuracy of detecting a “hit” we compared three cases, as explained in the following.

Case A: Action potentials were detected using an adaptive threshold (i.e., re-calculated at specific time intervals), and counted as an estimation of the global firing rate. We used spike counts in defined time intervals as features to identify the motor tasks. The firing rate was used as feature to classify either a “hit” or a “no-hit” (defined as an interval between two “hits”).

Case B: This case was similar to Case A, except that in this case we first utilized a wavelet transformation for denoising to improve the SNR of the data.

Case C: To compare the efficacy of Case A and Case B with the frequently used method in BCI research of manually setting the threshold levels for spike detection, we performed the MU spike detection without prior denoising the raw data and by using pre-determined thresholds registered by the experimenters at the beginning of each recording session (referred to as manual thresholding (MT) in the following).

As such, the data analysis consisted of the following steps (1) signal denoising with wavelets (only in Case B), (2) MU spike detection (using adaptive thresholding in Case A and B and MT for Case C), (3) feature extraction, (4) classification of the presence or absence of a “hit” using quadratic discrimination analysis, and (5) statistical evaluation of the classification results. All data analyses were carried out offline using Matlab (MathWorks, Natick, USA).

#### Signal denoising with wavelets

As a first step, we implemented a discrete wavelet transform to improve the SNR of the recorded intra-cortical signals. In brief, the signal was first transformed and decomposed into the wavelet domain, a threshold was applied to the wavelet coefficients to suppress the noise, and the denoised signal was transformed back to the time domain (a comprehensive description of the use of wavelets for denoising can be found in, e.g., Donoho, 1995; Diedrich et al., 2003; Kim and Kim, 2003). We selected 10 wavelets to be tested [Daubechie (2, 4, 6), Coiflets (2, 4, 5), Symlets (2, 4, 6), and Haar] mainly based on the resemblance between the wavelet shape and the shape of an AP. We applied a soft threshold (Donoho, 1995) and five decomposition levels. The effect and quality of the denoising considerably depends on the noise threshold (Donoho, 1995). In the present work, we used the following equation to estimate a threshold, Th, based on the noise level:

(1) $$Th=\gamma \sigma \sqrt{2\ln\left(N\right)}$$

where γ is a threshold correction factor, σ is the standard deviation of the noise estimated from the quantile–quantile plot, and *N* is the length of the data vector (Kamavuako et al., 2009). We tested 10 threshold levels, which were obtained by varying the threshold correction factor γ between 0.4 and 2.

To assess the effect of the wavelet denoising on the intra-cortical signal, we computed an estimate of the SNR in a 400 ms time window related to a “hit” (further specified in the paragraph below describing the feature extraction):

(2) $$SNR_{estim}=20\,\,\log_{10}\frac{A_{s}}{A_{n}},$$

where *A*_s_ is the maximum, absolute signal amplitude (also containing the noise), and *A*_n_ is the estimated noise amplitude calculated based on equation 3 below.

#### MU spike detection

In step 2 of the analysis the APs (or spikes) were detected using MT or adaptive thresholding.

In the case of the MT (Case C), the noise threshold level of each channel was visually estimated and registered once by the experimenters at the beginning of each recording session.

In the case of the adaptive thresholding (Case A or Case B), the detection threshold level, Thr_D_, was computed according to the equations described in (Donoho and Johnstone, 1993):

(3) $$Thr_{D}=4\sigma_{D}$$

(4) $$\sigma_{D}=\frac{median\left|x\right|}{0.6745},$$

where σ_D_ is the standard deviation of the data *x*. The duration of each data window was 400 ms. The estimation of the noise level was based on a median value to avoid the drawback of elevating the noise level due to high firing rates or high amplitude APs (Quiroga et al., 2004). We implemented a refractory period of 1 ms between detected spikes to minimize the number of false positives (Quiroga et al., 2004).

#### Feature extraction

In step 3 of the analysis, the task was to create and extract features from the intra-cortical responses so that we may later detect and classify whether a hit (referred to as “hit”) had occurred or not (referred to as “no-hit”). We based the selection of the “hit” time window on a study by Hyland and Jordan (1997) where it was demonstrated that muscle activity during reach tasks in rats initiates approximately 300 ms before the reach and continues up to 50 ms after. Thus, the cortical neural activity should precede any physical muscle activity. We based the selection of the “no-hit” time interval on visual observations during the experiment from which we concluded that this time window was a transition time for the rats to prepare to initiate a new hitting sequence. To incorporate information about temporal changes in the cortical neural activity, a “hit” was represented by spike counts in three 120 ms intervals (“*Int1*,” “*Int2*,” and “*Int3*”) up to -400 ms before the hit. A “no-hit” was correspondingly represented by three 120 ms intervals (“*Int1*,” “*Int2*,” and “*Int3*”) but up to -900 ms before the hit.

#### Classification

In step 4, the classification of the behavioral task was performed using a quadratic discrimination analysis, which has been widely used in BCI to classify multivariate data (Chapin, 2004). The quadratic discriminator maps the input features into a quadratic dimensional space and then uses a linear equation to classify the input data into different classes (Semmlow, 2009). For each trial of a session, the 3 spike counts in “*Int1*,” “*Int2*,” and “*Int3*” of the channels corresponding to paw movements were used as classification features. We implemented five-fold cross validation where 80% of the session’s data were used for training and the remaining data were used for testing.

#### Statistical analysis

As a final step, the outcome of the analysis was evaluated. The classification error was used to study the influence of the mother wavelet and threshold level. We further analyzed the classification error itself by defining a true positive (TP) classification as a “hit” that has been correctly classified as a “hit,” a false negative (FN) as a “hit” that has been misclassified as “no-hit,” a true negative (TN) as a “no-hit” that has been correctly classified as a “no-hit” and a false positive (FP) as a “no-hit” that has been misclassified as a “hit.” In each of the five folds, the fold classification error was calculated as the percentage of misclassified trials of a test set divided by the total number of trials in the test set, i.e., (FN + FP)/(TP + FN + TN + FP). Hence, the classification error was the average of the five folds’ classification errors. We calculated the sensitivity and specificity as TP/(TP + FN) and TN/(TN + FP), respectively, to evaluate the classifier’s performance. Generally, a good classifier is one that simultaneously has sensitivity and specificity values close to 100% (Semmlow, 2009). The accuracy of the classification was defined as 100% minus the classification error. A one-way analysis of variance (ANOVA) was utilized to compare the differences between the “no-hit” and “hit” classifications without denoising (Case A) and with denoising (Case B) and also to compare the differences between classification between Case B and C. The confidence interval of all statistical tests was 95%.

## RESULTS

### COMPARISON OF THE CORTICAL SIGNALS ENCODING A “HIT” AND A “NO-HIT”

**FIGURE 2:**
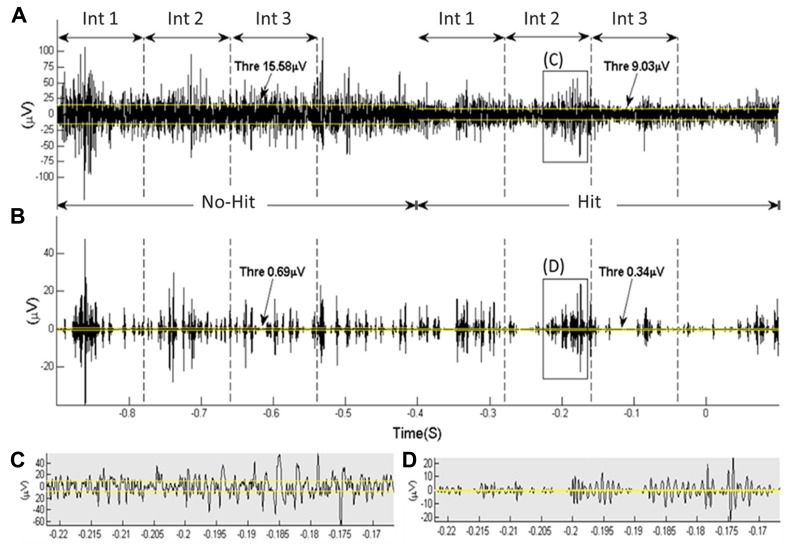
**An example of raw data obtained from one intra-cortical electrode’s channel from one rat.** The rat hit the paddle at 0 s. The time windows used to extract information about “hit” vs. “no-hit” classification (each is divided into three subintervals, “*Int1*,” “*Int2*,” and “*Int3,*” of 120 ms) are indicated. The yellow lines depict the noise threshold level (calculated over a 400-ms interval). **(A,B)** show the data before and after the wavelet denoising using Daubechie 6 and a denoising threshold factor (γ) of 0.8. **(C,D)** are close ups of selected data segments from **(A,B)**.

A visual, qualitative evaluation of the raw data showed differences in the cortical coding between a “hit” and “no-hit.” In **Figure 2**, an example of raw data is depicted with and without denoising (example data from one channel from one rat). Higher amplitude activity was typically observed during a “no-hit” window compared to during a “hit” window.

### CLASSIFICATION OF A “HIT” AND A “NO-HIT”

**FIGURE 3:**
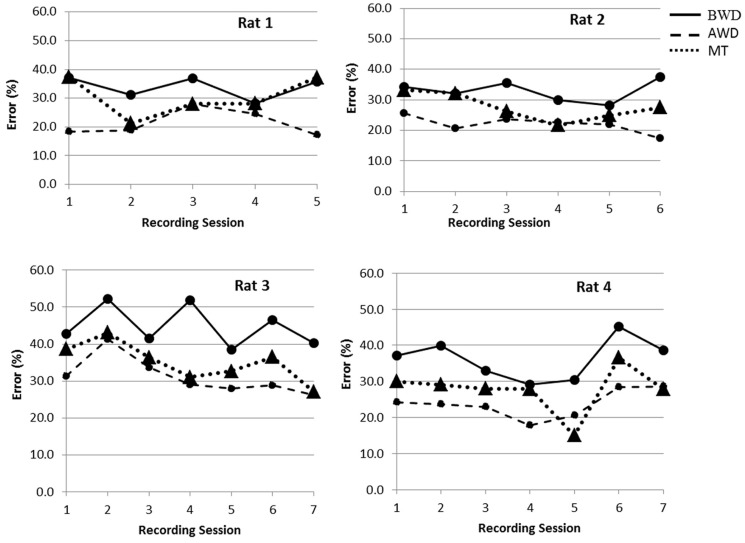
**Illustration of the error rate of a “hit” detection for each rat and each recording sessions (between five and seven sessions recorded on separate days).** The solid lines represent the classification error in *Case A* before wavelet denoising (BWD) while applying adaptive thresholds for spike detection. Dashed lines are the minimum classification errors in *Case B* that were obtained after the wavelet denoising (AWD). Dotted lines are classification errors in *Case C* using manual thresholding (MT) for spike detection without denoising the data.

We analyzed the influence of using adaptive thresholding without (Case A) and with (Case B) wavelet denoising on the accuracy of detecting the motor task. The classification of the behavioral task was performed by using the quadratic classification, and the results were validated with the five-fold cross validation. We found that the wavelet denoising had a positive effect on our ability to detect a “hit”. As such, the average classification errors across session decreased (Case A: rat 1 = 33.9 ± 4%, rat 2 = 32.9 ± 3%, rat 3 = 44.8 ± 5%, and rat 4 = 36.2 ± 5%, Case B: rat 1 = 21.3 ± 4%, rat 2 = 22 ± 2%, rat 3 = 31.2 ± 5%, and rat 4 = 23.8 ± 4%). This decrease was found to be statistically significant (*p* < 0.01). In **Figure 3**, the classification error found for each session is illustrated. In Case B, where wavelet denoising was applied, the minimum classification error obtained across wavelets and denoising thresholds are illustrated.

To evaluate the efficacy of applying adaptive thresholding we compared the results with the error of detecting motor tasks based on manually selected thresholds for spike detection (Case C). The average classification error across sessions in Case C were; rat 1 = 30.4 ± 6%, rat 2 = 27.6 ± 4%, rat 3 = 40.9 ± 5%, and rat 4 = 32.4 ± 6%). We found that the adaptive thresholding yielded a lower classification error in the majority of the cases. As such, adaptive thresholding with denoising (Case B) always provided the best classification, whereas adaptive thresholding without denoising (Case A) only performed better than MT (Case C) for two of four rats [in rat 1 and rat 2 the difference was significant (*p* í 0.04), in rat 3 and rat 4 there was no statistical significant difference (*p* ≥ 0.182)].

These findings were supported by calculating sensitivities and specificities. We found an average sensitivity of 77 ± 4% and an average specificity of 74.8 ± 6% after wavelet denoising (Case B). The same measures using MT (Case C) were found to be 73.3 ± 7% and 77.5 ± 6%, respectively, across all rats (see **Table 1**). We found the classification accuracy increased to 75 ± 6% after wavelet denoising (Case B) compared to 63.5 ± 4% without denoising (Case A), corresponding. In the case of the MT (Case C) the accuracy was 70 ± 6%.

**Table 1:** The sensitivities and specificities of the classification after wavelet denoising (Case B, AWD) or after using MT (Case C, MT).

	Sensitivity		Specificity	
	Case B (AWD) (%)	Case C (MT) (%)	Case B (AWD) (%)	Case C (MT) (%)
Rat 1	83 ± 3	62 ± 12	79 ± 5	76 ± 6
Rat 2	78 ± 7	76 ± 5	78 ± 5	69 ± 8
Rat 3	71 ± 6	73 ± 7	65 ± 6	79 ± 9
Rat 4	76 ± 4	82 ± 7	77 ± 5	86 ± 8

### EFFECT OF WAVELET DENOISING

To evaluate the effect of the wavelet denoising on the intra-cortical signals, we compared the SNR_estim_ of the “no-hit” and “hit” before and after denoising (see **Table 2**). The SNR_estim_ represents the average of the SNR_estim_ values computed from all combinations of mother wavelets and denoising thresholds. We observed a statistically significant increase in the SNR_estim_ as an effect of the denoising (*p* < 0.002 in all cases).

**Table 2:** Comparison of the average signal-to-noise ratios estimated for the cases of “no-hit” and “hit” before and after denoising.

	“No-hit” SNR_estim_ (dB)		“Hit” SNR_estim_ (dB)	
	BWD	AWD	BWD	AWD
Rat 1	10.6 ± 2.2	25.9 ± 3.6	7.8 ± 1.6	23 ± 2.6
Rat 2	10.2 ± 0.7	26.3 ± 1.2	6.8 ± 0.7	22.5 ± 0.9
Rat 3	8.4 ± 1.3	23.4 ± 2.1	6.4 ± 1.6	20.6 ± 2.2
Rat 4	7.1 ± 0.6	21.7 ± 0.8	5.4 ± 0.5	19.5 ± 0.8

*BWD stands for before wavelet denoising and AWD stands for after wavelet denoising.*

**FIGURE 4:**
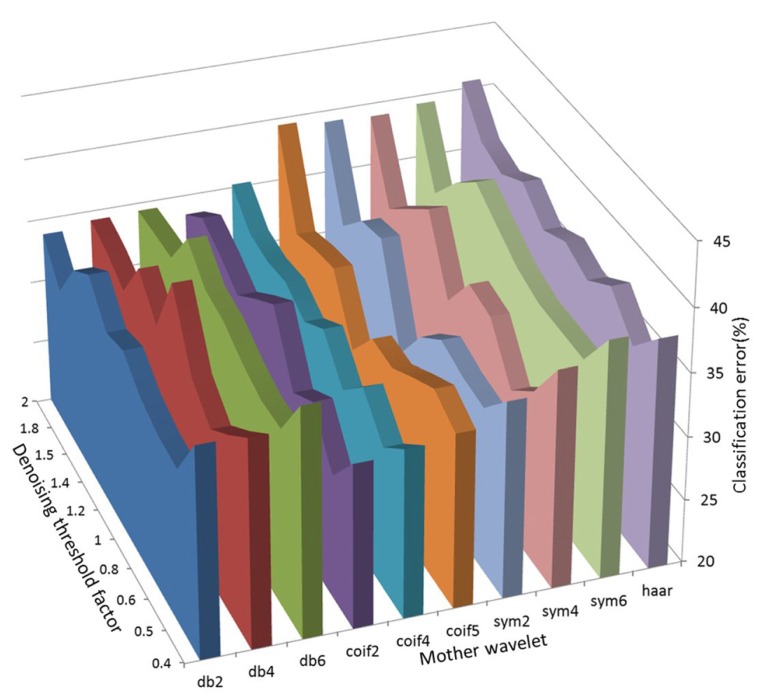
**A three-dimensional plot showing the average classification error for different combinations of the mother wavelet and denoising thresholds over seven recording sessions for one rat (rat 4)**.

We further investigated the effect of different combinations of mother wavelets and denoising thresholds to see if one particular combination could be chosen as the optimal. We found that each had a variable effect on the task classification from session to session and from animal to animal. As such, 23/25 recording sessions the combination of mother wavelet and denoising threshold that yielded the smallest classification error was unique. In **Figure 4**, a three-dimensional plot showing the average classification error for different combinations of the mother wavelet and denoising thresholds over seven recording sessions for one rat has been shown as an example (data from rat 4).

## DISCUSSION

In the present work, we investigated the possibility of detecting motor tasks from multi-units recordings in freely moving animal. We analyzed the influence of using adaptive thresholding for spike detection with and without prior denoising on the accuracy of detecting the motor task (referred to as Case A and Case B). We compared efficacy of our approach with the more traditional approach of detecting motor tasks based on, manually selected thresholds for spike detection (referred to as Case C). We found that using adaptive thresholding for spike detection together with wavelet denoising provided the highest classification rate.

### ON THE METHODOLOGICAL APPROACHES

The forelimb task consisted of paddle hit. As such, we decoded a type of “on–off” information, and no kinematics of specific limb positions was decoded. This decoding may be translated to a real-world application to detect the intention to activate an external device. We observed that the recorded amplitude activity was typically higher during a “no-hit” compared to during a “hit.” This is believed to be a result of the difference in motor behavior between the two cases. During a “hit,” the animal is more likely to only be moving one forelimb whereas in the “no-hit” case, the rat was more likely to be moving around in the cage or chewing.

We only included the channels that encoded “paw movements” (i.e., forelimb movements) during cortical microstimulation. The channels that revealed encoding of a mixture of “paw and neck movements” or “neck movements” were excluded because they likely included the contribution of neck-controlling neurons. The primary motor and somatosensory cortex areas, which represent forelimb movements and sensations, are partly overlapping in the rat and as such, “paw movement” responses may include somatosensory input originating from palmar contact with the paddle.

The majority of *invasive BCI*s reported in the literature rely on spike sorting to decode motor tasks (see e.g., Laubach et al., 2000; Wessberg et al., 2000; Donoghue, 2002; Musallam et al., 2004; Hu et al., 2005; Olson et al., 2005; He, 2008; Truccolo et al., 2008). However, it has been demonstrated that multi-unit data may also be used for accurate prediction and may be superior to spikes or local field potentials. For example, Stark and Abeles (2007) demonstrated a prediction accuracy of the hand movement direction and grasping of up to 90% based on MU recordings while the accuracy was only 70% using SU data.

Spike sorting requires complex signal processing to identify individual cells and separate them according to their waveforms. It has been estimated that 10–100 neurons are typically necessary to obtain accurate performance (Brown et al., 2004). This procedure requires time, power and, often, human intervention. Secondly, detection of SUs must be done frequently, since the recording of SUs is more prone to day-to-day variability.

In the current study, we detected the spikes and used that detection to count the occurrences of spikes in predefined windows. However, no additional spike sorting was performed in this study. This MU approach led to an approximately 75% correct classification in case B during free-moving tasks across the four rats included in the study. The results indicate the possibility of using *invasive BCIs* in less restricted environments, where the cortical signals may contain higher levels of background noise (such as noise from other biological sources) than what is typically found in classic laboratory conditions.

### EFFECT OF WAVELET DENOISING ON CLASSIFICATION OF A MOTOR TASK

We found that the SNR (i.e., comparison of calculated SNR_estim_values) of the data improved significantly by denoising the data using wavelets. As expected, the results showed also that different combinations of mother wavelet and denoising resulted in the lowest classification errors across rats and sessions and no unique or optimal combination could be identified *a priori*. The inconsistency in the selection of a particular combination of a mother wavelet and denoising threshold factor may be related to the daily variation of the data, i.e., if the number of APs that may appear in the recordings or the amplitude changes, this may cause different mother wavelets to be selected as the best match at different recording sessions. We found that the noise estimate threshold and/or the mother wavelet directly influenced the number of detected spikes. As such, if the number of detected spikes went down (i.e., very few spikes were detected), this corresponded to that less information was available to the classifier and the classification error increased. Our results support the fact that methods need to be developed to *a priori* identify and select this unique combination for each rat and each session as suggested in e.g., Kamavuako et al. (2010) and Shalchyan et al. (2012).

### EFFECT OF ADAPTIVE VS. MT FOR SPIKE DETECTION ON CLASSIFICATION OF THE MOTOR TASK

Our approach of using adaptive thresholding only yielded consistent lower classification error rates in the case where the data had first been subjected to denoising (Case B). Without prior denoising (Case A) the classification error rate was only better in two of four rats. As such, our results indicate that the classification accuracy may decrease if the adaptive thresholding is applied on noisy recordings. However, if the SNR of the recordings is increased with, e.g., wavelet denoising, as suggested in the present work, adaptive thresholding is a potential tool for developing an *invasive BCI* with minimal human interventions.

Stark and Abeles (2007) demonstrated that by using MU recordings from dorsal premotor cortex and ventral premotor cortex, reach and grasp may be predicted with ~90% accuracy. In another study, Fraser et al. (2009) demonstrated by using MU from the primary motor cortex the velocity of a hand movement could be accurately detected in 78 and 96% of the cases in two monkeys performing a standard center-out movement task. However, both studies were carried out under restricted lab conditions and experimenters set the threshold levels at the beginning of the recording session. The classification accuracy obtained in the present study is, as such, comparable.

Several decoding methods have been used in the past including linear (e.g., Kalman and Wiener filters), non-linear (e.g., support vector machines, SVMs and artificial neural networks, ANN), and statistical methods (e.g., Bayesian and hidden Markov models). In a previous study (Hammad et al., 2012), we reported on the use of an ANN and a support vector machine (SVM) to decode intra-cortical signals in a similar experimental paradigm, i.e., to detect if a “hit” had occurred. We found that the performance of ANN and SVM were comparable and also similar to the classification results obtained in the present study. The results indicate that the decoding methods perform equally well, and, as such, the quality of intra-cortical signals is of higher importance in the design of a robust and reliable *invasive BCI* system than the actual choice of the decoding method.

## CONCLUSION

We showed that a motor task can be detected with relatively high accuracy by analyzing multi-unit recordings from the M1 region of freely moving rats while applying adaptive thresholding. We also showed the positive impact of the wavelet denoising for improving the classification accuracy. However, our approach of using adaptive thresholding only yielded consistent lower classification error rates in the case where the data had first been subjected to denoising. For the future we recommend investigating the automatic selection of the wavelet and threshold for denoising. The work adds to the field on *invasive BCI* systems by demonstrating the possibility of exporting brain interfaces to less constrained conditions than in previous experimental paradigms.

## Conflict of Interest Statement

The authors declare that the research was conducted in the absence of any commercial or financial relationships that could be construed as a potential conflict of interest.
